# The Cell Wall of Seagrasses: Fascinating, Peculiar and a Blank Canvas for Future Research

**DOI:** 10.3389/fpls.2020.588754

**Published:** 2020-10-23

**Authors:** Lukas Pfeifer, Birgit Classen

**Affiliations:** Department of Pharmaceutical Biology, Pharmaceutical Institute, Faculty of Mathematics and Natural Sciences, Christian-Albrechts-University of Kiel, Kiel, Germany

**Keywords:** cell wall, seagrass, apiogalacturonan, sulfated polysaccharide, arabinogalactan-protein, lignin

## Abstract

Seegrasses are a polyphyletic group of angiosperm plants, which evolved from early monocotyledonous land plants and returned to the marine environment around 140 million years ago. Today, seagrasses comprise the five families *Zosteraceae*, *Hydrocharitaceae*, *Posidoniaceae, Cymodoceaceae*, and *Ruppiaceae* and form important coastal ecosystems worldwide. Despite of this ecological importance, the existing literature on adaption of these angiosperms to the marine environment and especially their cell wall composition is limited up to now. A unique feature described for some seagrasses is the occurrence of polyanionic, low-methylated pectins mainly composed of galacturonic acid and apiose (apiogalacturonans). Furthermore, sulfated galactans have been detected in some species. Recently, arabinogalactan-proteins (AGPs), highly glycosylated proteins of the cell wall of land plants, have been isolated for the first time from a seagrass of the baltic sea. Obviously, seagrass cell walls are characterized by new combinations of structural polysaccharide and glycoprotein elements known from macroalgae and angiosperm land plants. In this review, current knowledge on cell walls of seagrasses is summarized and suggestions for future investigations are given.

## Introduction

Around 140 million years ago, seagrasses evolved from early monocotyledonous land plants, which succeeded in conquering the marine environment. Today, they are a polyphyletic group of marine angiosperms with around 60 species in five families (*Zosteraceae*, *Hydrocharitaceae*, *Posidoniaceae, Cymodoceaceae*, and *Ruppiaceae*), which belong to the order Alismatales according to the Angiosperm Phylogeny Group IV System (APG IV, Chase et al., 2016). The genus *Ruppia*, which occurs in brackish water, is not regarded as a “real” seagrass by all authors and has been shifted to the *Cymodoceaceae* by some authors (Les and Tippery, 2013). The APG IV System and The Plant List Webpage (The Plant List, 2020) do not share this family assignment. We included *Ruppia* in this review to cover all literature with the connection to “seagrass” and used the traditional assignment to *Ruppiaceae*, resulting in five seagrass families. Seagrasses form important coastal ecosystems (Hemminga and Duarte, 2000). The worldwide endangering of these sea meadows, which provide food and habitat for many marine species, prompts the need for protection and understanding of these valuable resources. Recently, sequencing of the genomes of *Zostera marina* and *Zostera muelleri* allowed better understanding angiosperm adaption to the sea (Lee et al., 2016; Olsen et al., 2016). During the evolutionary step back to the ocean, different genes have been lost (e.g., stomatal genes) or have been reduced (e.g., genes involved in the synthesis of terpenoids) and others have been regained (e.g., genes involved in sulfation; Olsen et al., 2016). Genome information further revealed that adaption to the marine habitat was accomplished by severe changes of cell wall composition (Lee et al., 2016; Olsen et al., 2016). On the other hand, the cell walls of seagrasses are poorly understood. Beside ancestral traits of land plants, one would anticipate a habitat-driven adaption process to the new environment, which is characterized by multiple abiotic (high amounts of salt) and biotic (different seagrass grazers and bacterial colonization) stressors.

Although knowledge is limited, seagrass cell walls contain polysaccharides known from angiosperm land plants, e.g., cellulose (Syed et al., 2016). On the other hand, the cell walls of some seagrasses are characterized by sulfated polysaccharides (SP) (Aquino et al., 2005; Silva et al., 2012; Kolsi et al., 2016), a common attribute of the macroalgae from the groups of red, brown and also green algae. Recently the ability to synthesize SP was proposed to be regained by marine angiosperms (Aquino et al., 2005). Another unique feature of cell walls of seagrasses is the occurrence of unusual pectic polysaccharides called apiogalacturonans. Characteristic are high amounts of low-methyl esterified galacturonic acid (GalA*p*) units substituted with the unusual monosaccharide apiose (Api*f*) (Gloaguen et al., 2010; Lv et al., 2015).

In addition to polysaccharides, glycoproteins of the hydroxyproline-rich glycoprotein family (classified in Johnson et al., 2003), are important components of cell walls of land plants. The highly glycosylated arabinogalactan-proteins (AGPs) are interesting due to their involvement in both wall architecture and cellular regulatory processes (Ellis et al., 2010; Ma et al., 2018). AGPs are ubiquitous in seed land plants (Ma et al., 2018) and have also been found in ferns, lycophytes and mosses (Classen et al., 2019). They are structurally characterized by large polysaccharide moieties comprised of arabinogalactans (AGs, normally >90% of the molecule) which are covalently linked via hydroxyproline (Hyp) to relatively small protein/peptide backbones (normally around 1–10% of the molecule). The AGs of seed plants mainly consist of type II (3,6)-galactans with 3-, 6-, and 3,6-linked β-D-galactose (Gal*p*) residues, substituted with α-L-arabinose (Ara*f*) and often minor amounts of glucuronic acid (GlcA*p*) residues (Ma et al., 2018). Distinct glycan modifications have been identified in different species and tissues and are suggested to influence both their physical properties and function. Recently, AGPs have been isolated and structurally characterized for the first time from a seagrass (Pfeifer et al., 2020). Although the common backbone structure of land plant AGPs is conserved, the glycan structures exhibit unique features, including a high degree of branching and an unusually high content of terminating 4-O-methyl-glucuronic acid (4-OMe GlcA) residues, suggesting a role of seagrass AGPs in osmoregulation (Lamport et al., 2006).

Further components of secondary walls of plants are cross-linked phenolic polymers called lignin, which are responsible for mechanical strengthening of the wall. In seagrasses, this polymer has also been detected, but often in lower amounts compared to angiosperm land plants (Opsahl and Benner, 1993; Klap et al., 2000; Martone et al., 2009; Kaal et al., 2018).

Thus, cell walls of seagrasses seem to be fascinating combinations of features known from both angiosperm land plants and marine macroalgae with new structural elements. As dried seagrass leaves might be useful for papermaking or as insulating materials, knowledge on their cell wall composition is also important from a technological point of view. This review offers a detailed summary and discussion of literature on cell wall components of seagrasses.

## Polysaccharide Components of Seagrass Cell Walls

Table 1 gives an overview on isolation and characterization processes described for cell wall polysaccharides from seagrasses. Table 2 shows the already characterized polysaccharide structures from seagrasses.

**TABLE 1:** Overview on isolation and characterization processes for cell wall polysaccharides from seagrasses.

Species	Organ	Isolation	Structural analysis					References
			
			Monosaccharide composition	FT-IR	NMR	MS	Biological activities	
*Amphibolis antarctica*	Pollen grain	Enzyme containing buffer	√	–	–	–	–	Harris et al. (1994)
*Cymodocea nodosa*	Whole plant without root	Hot water after acetone and ethanol preextraction	√	√	√	√	√	Kolsi et al. (2016)
*Halodule pinifolia*	n.s.^1^	Hot water after depigmentation with acetone	–	√	–	–	√	Kannan et al. (2013)
*Halodule uninervis*	Leaves, rhizomes, roots	Sodium acetate buffer (10 mM), containing CaCl_2_ (3 mM) and adjusted to pH 5.0	√	–	–	–	–	Baydoun and Brett (1985); Waldron et al. (1989)
*Halodule wrightii*	n.s.^1^	Sodium chloride solution (0.25 M) adjusted to pH 8.0 after depigmentation with acetone	√	√	–	–	√	Silva et al. (2012)
*Halophila ovalis*	Leaves, rhizomes, roots	Sodium acetate buffer (10 mM) containing CaCl_2_ (3 mM) and adjusted to pH 5.0	√	–	–	–	–	Baydoun and Brett (1985)
*Halophila stipulacea*	Leaves, rhizomes, roots	Sodium acetate buffer (10 mM) containing CaCl_2_ (3 mM) and adjusted to pH 5.0	√	–	–	–	–	Baydoun and Brett (1985)
*Posidonia australis*	Leaves, whole plant	Hot sulfuric acid, 0.4 N; directly from plant material	√	–	–	–	–	Bell et al. (1954); Torbatinejad et al. (2007); Torbatinejad and Sabine (2001)
*Ruppia maritima*	Leaves, rhizomes, roots	Papain-containing buffer at pH 6.0	√	–	√	–	–	Aquino et al. (2005)
*Heterozostera tasmanica*	Root hairs	Washed cell walls directly hydrolyzed in 4% (w/v) sulfuric acid	√	–	–	–	–	Webster and Stone (1994)
*Phyllospadix torreyi*	n.s.^1^	Hot water, 5% ammonium oxalate, 7% sodium hydroxide	√	√	–	–	–	Woolard and Jones (1978)
*Zostera marina*	n.s.^1^; Leaves, rhizomes, roots	1% (w/v) aqueous ammonium oxalate with following pectinase treatment; aqueous extract and Yariv-precipitation after depigmentation with acetone	√	–	√	√	√	Gloaguen et al. (2010); Pfeifer et al. (2020)
*Zostera caespitosa*	n.s.^1^	Ammonium oxalate, 2% (w/v)	√	√	√	√	√	Lv et al. (2015)

*If more than one publication focused on one species, both are described in the “Isolation” column below each other. ^1^not specified plant organ used for extraction.*

**TABLE 2:** Structurally described charged polysaccharides from seagrasses.

Major monosaccharide components^1^	Linkages	References	Proposed structure
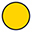	1,4- and 1,3-linked Gal, 2- and 4-O sulfated	Aquino et al. (2005)	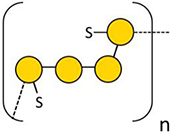

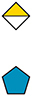	1,4-linked GalA; 1,2-linked Api	Gloaguen et al. (2010)	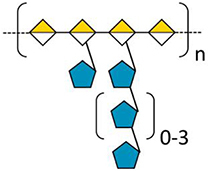

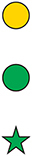	1,4-linked Gal, 6-O-sulfated	Kolsi et al. (2016)	not enough data

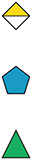	1,4-linked GalA; 1,3-linked Api	Lv et al. (2015)	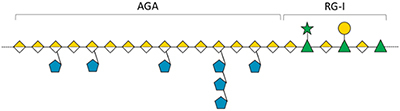

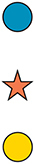	not determined	Silva et al. (2012)	not enough data

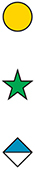	1,3-linked Gal; 1,4-linked GlcA; terminal Ara	Pfeifer et al. (2020)	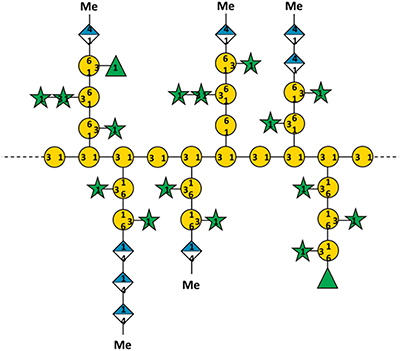

*Major polysaccharide components are symbolized using the Symbol Nomenclature for Glycans (SNFG) of the NCBI-Glycan Page (National Center for Biotechnology Information (NCBI), 2020). Abbreviations: Api, apiose; Ara, arabinose; Gal, galactose; GalA, galacturonic acid; and GlcA, glucuronic acid. ^1^monosaccharides are treated as “major” if they contribute more than 10% of the polysaccharide.*

### Cellulose

Comparable to angiosperm land plants, cellulose is a main component of seagrass cell walls. The content of cellulose has been determined in relation to dry plant material in different genera, e.g., *Halodule* (Waldron et al., 1989), *Halophila* (Baydoun and Brett, 1985; Waldron et al., 1989; Syed et al., 2016), *Posidonia* (Torbatinejad and Sabine, 2001; Torbatinejad et al., 2007; Khiari et al., 2010), *Zostera* (Davies et al., 2007), *Cymodocea*, *Enhalus* and *Thalassia* (Syed et al., 2016), and found to vary between 20% in *Posidonia australis* (Torbatinejad and Sabine, 2001) and 77% in *Enhalus acoroides* (Syed et al., 2016). A comparison between roots, leaves and rhizomes of *Halodule ovalis*, *Halodule stipulacea*, and *Halophila uninervis* revealed no obvious differences between the three species; in general, leaf tissue contained more cellulose compared to root and rhizome material (Waldron et al., 1989). It has to be taken into account, that the methods to determine the content of cellulose differ and are therefore difficult to compare (e.g., the method of Moubasher et al., 1982, is used to estimate cellulose, hemicellulose and lignin content in plant material). In general, the content of cellulose is estimated as the amount of insoluble material after hydrolysis. The methods for hydrolysis vary and furthermore, the insoluble residue is sometimes regarded as cellulose content, sometimes the carbohydrate part of the insoluble residue is determined by the photometric method of DuBois et al. (1956). Whereas in some cases only TFA (2 N) is used for hydrolysis (Waldron et al., 1989), other authors perform TFA (2 N) treatment first followed by further hydrolysis with concentrated sulfuric acid (Davies et al., 2007). In both cases, the sugar content in the insoluble residues is determined photometrically to give the cellulose content. Other authors use alkaline treatment with KOH (residue A: cellulose and lignin), followed by hydrolysis with strong sulfuric acid (residue B: lignin) and calculate the cellulose content (A–B) without determination of the carbohydrate content (Syed et al., 2016). In none of the literature reviewed, the type of cellulose (α- or β-cellulose) was determined. To gain more information on that, it is recommended to perform a fractionated extraction (Galiwango et al., 2019) and use the different solubility of both types in alkaline conditions.

### Hemicelluloses

Hemicelluloses are a large family of cell wall polysaccharides including different polymers. Syed et al. (2016) estimated the amount of hemicelluloses in different seagrasses using the method of Moubasher et al. (1982). The values ranged from 14 – 28% of dried seagrass material (*Thalassia hemprichii* 14%, *Halophila spinulosa* 23%, *Cymodocea serrulata* 26%, *Halophila ovalis*, and *Enhalus acoroides* 28%). For *Posidonia australis* (Torbatinejad et al., 2007; Torbatinejad and Sabine, 2001) and *Posidonia oceanica* balls (Khiari et al., 2010) the amounts were 11.7 and 21.8%, respectively. Highest values for hemicelluloses were determined for *Z. marina* (38%; Davies et al., 2007).

#### Xylans

Xylans are a group of plant cell wall polysaccharides with a backbone consisting of 1,4-linked β-D-xylopyranoses, which have a high degree of substitution (York and O’Neill, 2008; Hatfield et al., 2016; Peña et al., 2016; Tryfona et al., 2019). Xylans are the main hemicelluloses in the cell walls of most plant species (Tryfona et al., 2019) and play an important role in crosslinking with other structural components (cellulose, lignin). In primary cell walls of plants belonging to the *Poales* (also monocotyledonous plants), xylans are present in high amounts.

Most of the publications on seagrass xylans are based on crude monosaccharide quantification; investigations on exact structures of seagrass xylans are missing. Baydoun and Brett (1985) could show that significant quantities of xylose and arabinose were present in non-cellulosic polysaccharide fractions of *Halophila stipulacea* and *H. ovalis* and attributed this to presence of arabinoxylans. In the same study, *Halodule uninervis* showed only small amounts of xylose in the same cell wall fraction. In support of this finding, Brudecki et al. (2015) measured a low xylan content of around 5% for cell walls of *H. uninervis* with similar methods. On the other hand, *Z. marina* fibers contain 38% hemicelluloses, which are mainly xylans (Davies et al., 2007). Are they complex heteroxylans like in grasses or more similar to algal 1,4-linked- (charophytic green algae), 1,3-linked- (chlorophytic green algae/some red algae), or 1,3; 1,4-linked-homoxylans (red algae) (Hsieh and Harris, 2019)? The next step forward to answer this question would be a broader investigation: Modern methods like xylan epitope profiling (Peralta et al., 2017), capillary electrophoresis based high-throughput carbohydrate profiling (Li et al., 2013), solid-state NMR methods (e.g., Dupree et al., 2015) or HILIC-MALDI-ToF/ToF-MS/MS (Busse-Wicher et al., 2016) have to be carried out to give insights in xylan structure of seagrasses.

#### Mannans

Mannans are important members of the hemicellulose family, which are subdivided into linear mannans, galacto-, gluco- and galactoglucomannans (Petkowicz et al., 2001; Moreira and Filho, 2008). They have mainly structural functions, but also signaling functions are proposed (Moreira and Filho, 2008). They are present in eukaryotic algal species belonging to the divisions of Rhodophyta and Chlorophyta, where they seem to replace cellulose as the main cell wall carbohydrate (Painter, 1983). To the best of our knowledge, there are no described mannans in any seagrass species. He et al. (2015) postulated a correlation of mannan accumulation in *Dendrobium officinale* with water deficiency stress. Even though *D. officinale* is not a marine plant, this phenomenon could be also apparent in seagrass species. More studies with a focus on mannans are necessary to answer the question whether mannans are components of seagrass cell walls.

#### Xyloglucans

Xyloglucans are found in primary cell walls of all angiosperms, where they are responsible for the crosslinking of cellulose microfibrils (Popper and Fry, 2008; Brennan and Harris, 2011). They consist of 1,4-linked β-D-glucose residues which form a backbone substituted with α-D-xylosyl chains at *O*-6. Xyloglucans with the special side chain α-L-Fuc*p*-(1,2)-β-D-Gal*p*-(1,2)-α-D-Xyl*p*-(1,6)-β-D-Glc*p* are called fucogalactoxyloglucans and are common in primary walls of non-commelinid monocotyledons, while they are rare in commelinids. In a study with the monoclonal antibody CCRC-M1, which recognizes the epitope structure α-Fuc-(1,2)-β-Gal, *Zostera muelleri* interestingly differs in its fucogalactoxyloglucan composition from the other investigated non-commelinid monocotyledons. While most of the investigated non-commelinid species showed a widely distributed fluorescence labeling with this antibody comparable to *Arabidopsis thaliana*, *Z. muelleri* was labeled only in the phloem sieve elements (Brennan and Harris, 2011).

The low amount of fucogalactoxyloglucan in *Zostera muelleri* (Brennan and Harris, 2011) is supported by the fact that in the genome of another *Zostera* species there were only 2 GT37 genes, which encode the xyloglucan fucosyltransferases (Olsen et al., 2016). In contrast to that, *Arabidopsis* or *Oryza* have 10 or 18 of these enzyme genes, respectively.

#### Mixed-Linked Glucans

The so-called mixed linked glucans (MLG), consisting of β-D-glucose chains connected through 1,3- and 1,4-linkages, have been found in high amounts in the Poales order (Popper and Fry, 2004), in *Centraria islandica* (as “lichenan;” Stone and Clarke, 1992), in the genus *Equisetum* (Fry et al., 2008) and most recently in *Phaeophyceae* cell walls (Salmean et al., 2017). In the *Poales* order MLG is present at certain stages during the primary wall formation in maize (Penning et al., 2019). Baydoun and Brett (1985) as well as Waldron et al. (1989) attributed the high amounts of glucose in *Halodule uninervis* to the presence of MLG. Structure elucidation has to be performed to prove this proposal.

### Pectic Polysaccharides

Pectin is a structurally complex carbohydrate family rich in galacturonic acid, including the major polysaccharides homogalacturonan, rhamnogalacturonan I, rhamnogalacturonan II and xylogalacturonan (for an in-depth review, see Mohnen, 2008). Their characteristically high amount of acidic domains is important for the osmotic properties and can interact with ions and low-molecular-weight compounds (Willats et al., 2001).

In some seagrasses, a special pectic polysaccharide named “apiogalacturonan” is present (Table 2). Miroshnikov (1940) first isolated an uronic acid rich polysaccharide with gelforming properties from *Z. marina*, which he named “zosterine.” Analytical characterization of zosterine was broadened by the studies of the group of Ovodova and Ovodov (e.g., Ovodova et al., 1968; Ovodov et al., 1971a,b, 1975; Popov et al., 2007), which detected D-apiose as substantial monosaccharide of this pectic fraction.

Recent studies (Gloaguen et al., 2010; Lv et al., 2015) investigated the fine structure of apiogalacturonans in two different *Zostera* species after extensive purification steps. Its structure was analyzed with modern instrumentation (enzymatic digestion + mass spectrometry of fractions; one- and two-dimensional NMR experiments). The described structures consist of an α-1,4-linked-D-galacturonan substituted at position C-2 or C-3 of GalA by single apiose residues or short oligosaccharides of apiofuranose (Table 2).

Apiogalacturonans of seagrasses have a very low degree of esterification around 10% (Maeda et al., 1966; Khotimchenko et al., 2012). Recent genetic studies of two *Zostera* species (Lee et al., 2016; Olsen et al., 2016) revealed an increase in unique pectin methylesterase-related domains in different proteins possibly responsible for the low degree of methyl-esterification. It is hypothesized that this represents a control mechanism for osmoregulation. Furthermore, investigations on heavy metal binding by apiogalacturonans (e.g., cerium and mercury, Khotimchenko et al., 2006, 2012; cadmium and lead, Khozhaenko et al., 2016) might help to understand biophysiological functions of apiogalacturonans in “high-charge environments” like the marine habitat.

Arabinans are composed of a α-1,5-linked arabinofuranose backbone and may occur separately in the wall or as neutral side chains of rhamnogalacturonan-I (Wefers and Bunzel, 2016). Although an “arabinan” content has been described for *Halodule uninervis* (Brudecki et al., 2015) only a very crude compositional analysis was performed in this investigation. Whether seagrasses contain arabinans is therefore unknown to date and needs to be investigated in more detail (e.g., by a fast approach with a combination of two-dimensional NMR spectroscopy with previous enzyme-assisted extraction, like established by Wefers and Bunzel, 2016).

### Sulfated Polysaccharides

Sulfated polysaccharides have been found in a number of marine organisms (e.g., Pomin, 2012; Ngo and Kim, 2013) and are often described as compounds with a number of bioactivities, including antioxidant, anticancer and anticoagulant activities (Ngo and Kim, 2013). Their occurrence is mostly correlated with a saline environment (Aquino et al., 2011).

**FIGURE 1:**
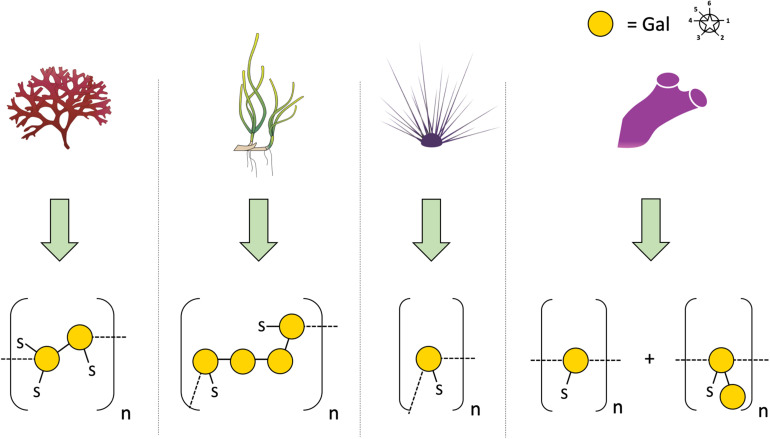
Diverse structures of sulfated galactans from marine organisms. Sulfated polysaccharide structures from left to right: red algae: *Botryocladia occidentalis*, seagrass: *Ruppia maritima*, sea urchin: *Echinometra lucunter*, tunicate: *Styela plicata*. Illustration adapted from Aquino et al. (2005). All vector images are from Tracey Saxby, Jane Thomas, Jane Hawkey, IAN Image Library (ian.umces.edu/imagelibrary/).

In 2005, SP were first detected in the seagrasses *Halodule wrightii* and *Halophila decipiens* as well as in the marine angiosperm *Ruppia maritima* (Aquino et al., 2005) in amounts around 1% (m/m). Structure elucidation was performed only for the galactan from *R. maritima* and revealed a 2-*O*- and 4-*O*-sulfated galactan, which consists of a repeating tetrasaccharide [3-β-D-Gal-2(OSO_3_)-1,4-α-D-Gal-1,4-α-D-Gal-1,3-β-D-Gal-4(OSO_3_)] (Table 2 and Figure 1). In an additional study from the same working group (Aquino et al., 2011) it was shown, that the amount of this SP increased in higher salinity and disappeared in culture without salt supplement.

Silva et al. (2012) postulated the presence of a sulfated heteropolysaccharide in *H. wrightii*, consisting of glucose:xylose:galactose (1:1:0.9) with a sulfation degree of 20% and a molecular weight of ∼11 kDa. As the sample was treated with trichloroacetic acid (10%) during the isolation process (Table 1), which is, to the best of our knowledge, a polysaccharide-degrading and sulfate-deleting condition, the presence of this SP in *Halodule* has to be verified by further investigations.

Occurrence of a “fucoidan-like sulfated polysaccharide” has been proposed for *Halodule pinifolia* (Kannan et al., 2013). The structural data has to be regarded as preliminary as it based only on Fourier-Transformation infrared spectroscopic (FT-IR) analysis and colorimetric total sugar determination.

A sulfate containing polysaccharide fraction has been isolated from *Cymodocea nodosa* by water-extraction followed by ethanol-precipitation (Kolsi et al., 2016). Structural analytics (NMR, LC-ESI-MS) led to the proposal of a branched 1,4-linked galactosidic backbone with 6-O-sulfation and decoration with small amounts of other monosaccharides. Due to the isolation procedure (see above), the sulfated galactan is most likely accompanied by other water-soluble polysaccharides.

### Callose

Callose is a homopolysaccharide composed of β(1,3)-linked glucose with some β(1,6)-branches which is typically found in sieve plate pores, in plasmodesmata, in the cell plate during cell division and also in pollen (Wu et al., 2018). It is synthesized by callose synthase (Verma and Hong, 2001) and plays an important role in biotic and abiotic stress response (Lampugnani et al., 2018). An easy method to detect callose in light microscopy is anilin blue fluorochrome staining of plant tissue. Whereas the pollen walls of *Amphibolis antarctica* showed no staining with anilin blue (Ducker et al., 1978; Harris et al., 1994), callose staining was positive between daughter cells in the pollen tetrads of *T. hemprichii* and *T. ciliatum*. Callose was also detected by this staining in pollen of *H. wrightii* and *Z. marina* (Pettit and Jermy, 1975). As reaction with this dye is not exclusive for callose (Smith and McCully, 1978), more specific methods for detection should be applied like e.g., the use of the monoclonal antibody AB 400-2. With this antibody, detection of β(1,3)- in the presence of β(1,4)- or β(1,3)-β(1,4)-linked glucan structures is possible (Meikle et al., 1991) and it was also used for immunolocalization of callose in streptophyte green algae (Herburger and Holzinger, 2015). A hint of the general ability of at least some seagrasses to synthesize callose is the presence of “Glucan synthase-like 8” genes in *Halophila* and two *Zostera* species (Lee et al., 2018). In *Arabidopsis*, these genes encode enzymes responsible for callose synthesis.

## Wall (Glyco)Proteins

As Johnson et al. (2003) proposed, the term “wall protein” is used in this review to refer to all types of glycosylated proteins, despite their degree of glycosylation or protein-glycosylation type. The literature about this group of biomolecules from seagrasses is mainly limited to some works on pollen cell walls. Ducker and Knox (1976) first proposed the presence of specialized “glycoproteins” in pollen cell walls of *Amphibolis antarctica* as a unique adaption to be able to fulfil submarine pollination sufficiently. Based on this hypothesis Pettitt (1980) performed microscopy of *T. hemprichii* fresh-frozen pollen with β-glucosyl Yariv (βGlcY) – a reagent used for selective interaction with AGPs. AGPs, which are a group of highly glycosylated (*O*-linkage *via* hydroxyproline) proteins are involved in many plant cell wall functions and processes (Ellis et al., 2010). *T. hemprichii* pollen walls showed positive interaction with βGlcY. In discrepancy to that, Harris et al. (1994) performed extraction of *Amphibolis antarctica* pollen walls and concluded absence of hydroxyproline-rich glycoproteins – a group of wall proteins, including the extensins, AGPs and proline-rich proteins. “Small amounts of glycin-rich proteins […] may be present” (Harris et al., 1994). These assumptions were based on monosaccharide and amino acid composition of acidic wall preparations. Pfeifer et al. (2020) performed the first isolation and structural characterization of a βGlcY-precipitable AGP from *Z. marina* (Table 2). Here whole plants, rhizomes, roots and leaves were extracted and investigated. In addition to that microscopic data and thermodynamic binding analysis showed that unique 1,4-linked- and terminal-glucuronic acids in this AGP fraction showed calcium binding properties with a *K*_D_ value in a micromolar range. This observation underlines the proposed functionality of AGPs in salt adaption (Lamport et al., 2006). A broader investigation on presence in other seagrass species could be of scientific value, especially in the light of independent adaption to the sea in minimum three seagrass lineages (Williams, 2016).

As far as *N*-linked glycosylated wall proteins are concerned, the only work on a seagrass was done by Yoshiie et al. (2012) showing that *Z. marina* contains high-mannose type *N*-glycans in high amounts. These N-linked glycans also possess a wide range of functions intensively discussed by Strasser (2014).

## Lignin

Beside the polysaccharide components, the secondary walls of vascular land plants consist of condensed macromolecules with cross-linked phenolic monomers, called lignin. These polymers are described as containing about 30% of the organic carbon in biosphere (Boerjan et al., 2003). Due to their molecular structure, their ability to covalently complex with wall polysaccharides and their tendency to encrust cellulose microfibrils, they contribute to increasing hydrophobia in secondary plant walls which results in dehydration. An effect of this process is the gain of mechanical strength and reduction of flexibility for the tissue and for the whole plant (Doblin et al., 2010; Weng and Chapple, 2010).

For a long time, it was not clear whether seagrasses commonly have lignins as cell wall components, because it was hypothesized that due to the experimental methodology other phenols, being present in many seagrass tissues (Lewis and Yamamoto, 1990), could appear as “lignin.” Therefore, it was stated that “evidence for lignin in submerged aquatic plants is not convincing. More definitive proof is necessary if these are to be considered lignin-synthesizing organisms” (Lewis and Yamamoto, 1990). Furthermore, there is no consistency in the methodologies of the existing literature. On the other hand, investigation of two seagrass species (*Posidonia oceanica* and *Z. marina*) and different tissues of these with Curie-point pyrolysis gas chromatography mass spectrometry (Py-GCMS) revealed that lignin is present in these seagrasses with variations between species and tissues (Opsahl and Benner, 1993; Klap et al., 2000). The retention of lignin during evolution from terrestrial vascular plants back to the sea might be as a useless artifact or an evolutionary advantage also in marine environment. The first idea is questionable due to the recently described occurence of lignin or lignin-like macromolecules in red algae and aquatic green algae (Martone et al., 2009). The second proposal is more conclusive. This topic is discussed intensively by Klap et al. (2000), who found that rhizomes of seagrasses are richer in lignin compared to leaves and suggested that lignification contributes to the longevity of a tissue by protecting it against microbial attack.

According to Syed et al. (2016) the amount of lignin in species of the genera *Enhalus*, *Cymodocea*, *Halophila* and *Thalassia* is low with 5–11% of dry weight. In that work, the insoluble residue after treatment with 24% potassium hydroxide followed by strong sulfuric acid was quantified as lignin.

**FIGURE 2:**
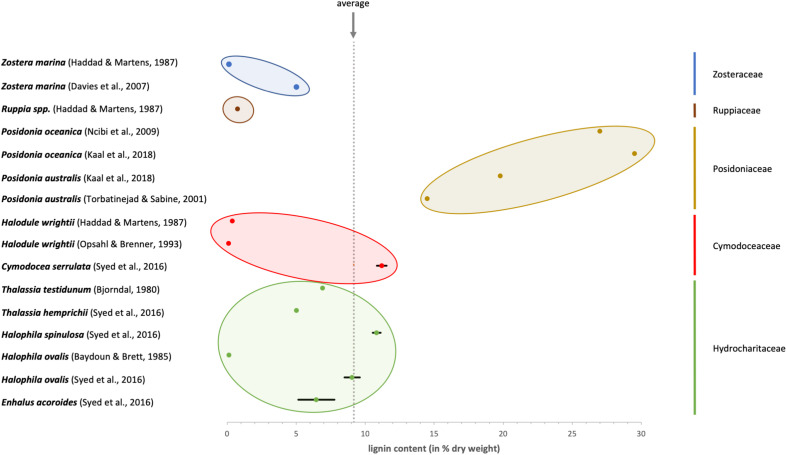
Overview about the published values for lignin content in different seagrass species. The colors identify the membership to the five systematic families of seagrasses.

Looking at the published data (see Figure 2), it seems as if the species belonging to the *Posidoniaceae* contain more lignin compared to all the other families (*Cymodoceaceae*, *Hydrocharitaceae*, *Ruppiaceae*, and *Zosteraceae*) which can be regarded as low in lignin content. It has to be taken into account that *Posidoniaceae* species are responsible for extensive detritus production. As exact definition of material used is missing in some publications, results might be influenced by degraded materials, which contain higher amounts of lignin. Therefore, results on *Posidonia* balls (e.g., Khiari et al., 2010), which are not comparable to the fresh *Posidonia* material, were excluded from Figure 2.

Knowledge about lignin is also of interest, because recent publications on blue carbon stocks (Barry et al., 2018; Serrano et al., 2020) showed need of lignin data for their calculations.

## Information on Cell Wall Relevant Genes

With the increasing field of -omics studies more and more data is accessible, which can be used for nearly each kind of comparison between marine angiosperms and other plant groups or in the polyphyletic group of seagrasses itself. Up to now two full genome sequences have been published. The authors compared the genomes of *Z. marina* (Olsen et al., 2016) or *Z. muelleri* ssp. *capricornii* (Lee et al., 2016), respectively, with the only other sequenced relative from the order of Alismatales, *Spirodela polyrhiza* and in addition to that to some angiosperm land plants. These two publications gave insight into the molecular adaptation steps to marine environment and supported that severe changes in cell wall composition were necessary.

Sablok et al. (2018) provided access to a transcriptomics database called “SeagrassDB”, which allows authors to perform comparative trancriptomics on eight seagrass species and other aquatic plant species. With the same idea Wissler et al. (2009) implemented the “Dr. Zompo” database, which is focused on *Z. marina* and *Posidonia oceanica*. Both resources could enable specialists on different cell wall components to test their hypotheses, regarding for example the variety of carbohydrate active enzymes (Table 3).

**TABLE 3:** Enzymes with activities in biosynthesis of the different cell wall components.

Cell wall component	Species	Enzymes^∗^	References
Cellulose	*Z. marina*	12 CESA; 4 CSLG	Olsen et al. (2016)
Xylans	*Z. marina*	9 GH3; 3 GH10; 1 GH51; 39 GT8; 10 GT14; 36 GT47; 13 GT61; 1 CE6	Olsen et al. (2016)
Mannans	*Z. marina*	6 CSLA; 3 CSLD; 4 GT5_7	Olsen et al. (2016)
Xyloglucans	*Z. marina*	3 CSLC; GH16; 5 GT34; 2 GT37	Olsen et al. (2016)
	*Zostera*	Xyloglucan endotransglucosylase/hydrolase 5	Lee et al. (2018)
Mixed-linked glucans	*Z. marina*	absent	Olsen et al. (2016)
Pectins	*Z. marina*	39 GT8; 36 GT47; 56 GH28; 16 PL1; 63 CE8	Olsen et al. (2016)
Sulfated polysaccharides	*Z. marina*	to be further investigated	Olsen et al. (2016)
Callose	*Z. marina*	19 CBM43; 40 GH17; 10 GT48;	Olsen et al. (2016)
	*Zostera*	Glucan synthase-like 8	Lee et al. (2018)
Wall (glyco-)proteins	*Z. marina*	4 DUF579; 14 GT14; 25 GT31; 4 GT61; 13 GT77	Pfeifer et al. (2020)
Lignin	*Z. marina*	not investigated	

*^∗^Activities were used as stated in the respective reference.*

For a detailed overview about molecular profiling and – omics techniques with a full list of all accessible data on seagrasses until the year 2016, see Davey et al. (2016).

## Technical Applications

Large amounts of different seagrasses wash on beaches of the world each year, sometimes causing even environmental problems. Therefore, an economic use of this resource would be desirable. For *Posidonia australis*, use as foodstuff for ruminant animals has been proposed (Torbatinejad et al., 2007). Another possible application of seagrass material is use as source of fibres. Today, pulp production from non-wood material increases, sometimes due to shortage of hard-wood fiber material (Saijonkari-Pahkala, 2008). Use of seagrass material for papermaking has been evaluated for different seagrass species, e.g., *Z. marina* (Davies et al., 2007), *P. oceanica* (Khiari et al., 2010), *E. acoroides*, *C. serrulata*, *H. ovalis*, *H. spinulosa*, and *T. hemprichii* (Syed et al., 2016). The comparable low lignin content is an advantage as bleaching costs are lower. Among the species investigated by Syed et al. (2016), *Enhalus acoroides* was most preferable for papermaking because of the highest cellulose content combined with the longest fibers. Another interesting feature of dry seagrass material is its suitability as environmentally friendly, fire-resistant insulating material. Already at the beginning of the 20th century, wild gathering of the leaves of *Z. marina* formed the basis of an insulation industry in North America (Wyllie-Echeverria and Cox, 1999). Today, there are efforts to revive this industry, e.g., *Posidonia oceanica* balls are gathered and sold as insulating material. Their thermophysical behavior seems to be very promising for this purpose and only a few pre-treatment steps with sodium hydroxide could enhance the properties of *Posidonia* fibers to a level comparable to industrial insulating materials (Hamdaoui et al., 2018). Another approach for construction purposes has been registered for patent in 2018 (Pavlakis, 2018). The patent holder sells environmentally friendly panels build from *Posidonia* seagrass balls. Furthermore, the combination of a relatively high amount of cellulose and hemicelluloses together with a low lignin content makes seagrasses an interesting starting material for the production of biofuel. For this kind of use lignin usually has to be removed to enable successful chemical or enzymatic degradation in acceptable yields (Mukherjee et al., 2016). This approach was proposed for *Z. marina* detritus in Turkey (Ncibi et al., 2014).

## Conclusion and Perspectives

### Conclusion With Regard to Existing Work

Despite the ecological importance of seagrasses, which form important coastal ecosystems worldwide, the seagrass cell wall as a whole is poorly understood. The content of cellulose, hemicelluloses and lignin is often estimated by simple procedures using different hydrolytic methods and weighing of the residual material. On the other hand, some detailed investigations of single seagrass polysaccharides in single species shed light on some aspects of seagrass cell wall adaption to the marine habitat (e.g., Aquino et al., 2005; Gloaguen et al., 2010; Lv et al., 2015; Pfeifer et al., 2020). All together, similarities and differences between the different seagrass families and species and also the comparison to cell walls of monocotyledonous land plants are still a mystery.

Especially the hemicelluloses are poorly investigated up to now. Although a content of over 30% in relation to dry plant material has sometimes been estimated for these polysaccharides, most publications comprise only rough determinations of components without in-detail structural elucidations like it is state-of-the-art in this particular field of cell wall research. For technical applications, knowledge on cellulose content and nature of hemicelluloses are necessary to evaluate the suitability of seagrass material for papermaking or production of biofuels.

With regard to pectic polysaccharides, the presence of apiogalacturonans not known from land plants has been shown for two *Zostera* species (Gloaguen et al., 2010; Lv et al., 2015). Up to now it is unknown whether apiose-rich carbohydrates are only limited to a few seagrass species or a general feature, being relevant for angiosperm life in the marine habitat.

Another unique feature of seagrass cell walls might be the presence of SP. A sulfated galactan from *Ruppia maritima* has been isolated and carefully characterized (Aquino et al., 2005). Although there are some proposals for the presence of other SP in seagrasses (e.g., Aquino et al., 2011; Silva et al., 2012; Kolsi et al., 2016), proof of sulfate by colorimetric assays or FT-IR is not sufficient, as seagrasses contain other sulfated compounds like e.g., zosteric acid or sulfated flavonoids (Zidorn, 2016). According to Olsen et al. (2016), an expansion of arylsulfotransferases in *Zostera* compared to land plants might correlate with the ability to synthesize SP. Careful evaluation whether SP are a general feature of seagrasses is urgently needed.

In the field of wall proteins, the recent findings from our group (Pfeifer et al., 2020) could underline the hypothesis of an involvement of highly charged AGPs in calcium storage and – signaling (Lamport et al., 2006; Lamport et al., 2020). To answer the question whether AGPs are present in other seagrass species and whether they are structurally comparable to *Zostera* AGP is another challenge for the future.

Although a high number of literature on lignin in seagrasses is available, more definite proof with modern methodologies, like Py-GC-MS (e.g., procedure of van Erven et al., 2017) or qNMR (e.g., procedure of Capanema et al., 2005) is necessary with a focus on more species from all seagrass families.

### Main Challenges for the Future

At first, comprehensive approaches on evaluation of seagrass cell wall composition are needed to understand the general composition of the cell walls of these angiosperms living in an extreme environment. This could be done by sequential extraction of seagrass material following published protocols (e.g., O’Rourke et al., 2015; Raimundo et al., 2016) and analytical characterization of the different cell wall fractions. To get information on similarities and differences between the cell walls of different seagrass families, so many species as possible, belonging to the different seagrass families, have to be investigated. It has to be taken into account, that the availability of seagrass material is often limited due to the strict rules for protection of seagrasses in their natural habitats.

Furthermore, it has to be investigated whether the interesting unique polysaccharides/glycoproteins which have been isolated and carefully characterized for single seagrass species are also present in other or even all seagrasses. Especially the apiogalacturonans (Gloaguen et al., 2010; Lv et al., 2015), sulfated galactans (Aquino et al., 2005) and highly charged AGPs (Pfeifer et al., 2020) are interesting candidates for adaption to the marine habitat. Comparable to marine algae, charged polysaccharides/glycoproteins of the cell wall seem to be essential to cope with salt stress. A future task will be to elucidate the adaption strategies of the different seagrass lineages that evolved to marine environment independently. Furthermore, the cellular mechanisms involved in protection against salt have to be investigated. Both sulfate groups and uronic acids are able to bind Ca^2+^ ions, which play a crucial role in both the regulation of transport and exclusion of Na^+^ and other mineral ions at the plasma membrane of plant cells and are able to protect a salt-sensitive species (*Phaseolus vulgaris*) against damage caused by NaCl present in the cell culture medium (Lahaye and Epstein, 1969). For AGPs of *Z. marina*, a strong binding of Ca^2+^ has already been shown by bio-layer interferometry (Pfeifer et al., 2020).

Finally, full genome sequencing of more seagrass species is necessary to get widespread information on cell wall related genes of these fascinating organisms. Identification of seagrass genes involved in adaption of cell walls to salt water could provide information how agricultural crops might tolerate an increasingly dry and saline environment.

## Author Contributions

LP and BC performed literature search and evaluated the published data. LP created tables and figures. Both authors discussed the results and wrote the final manuscript. Both authors contributed to the article and approved the submitted version.

## Conflict of Interest

The authors declare that the research was conducted in the absence of any commercial or financial relationships that could be construed as a potential conflict of interest.

## References

[B1] Aquino R. S. Grativol C. Mourão P. A. S. (2011). Rising from the Sea: correlations between sulfated polysaccharides and salinity in plants. *PLoS One* 6:e18862. 10.1371/journal.pone.0018862 21552557 PMC3084243

[B2] Aquino R. S. Landeira-Fernandez A. M. Valente A. P. Andrade L. R. Mourão P. A. S. (2005). Occurrence of sulfated galactans in marine angiosperms: evolutionary implications. *Glycobiology* 15 11–20. 10.1093/glycob/cwh138 15317737

[B3] Barry S. C. Bianchi T. S. Shields M. R. Hutchings J. A. Jacoby C. A. Frazer T. K. (2018). Characterizing blue carbon stocks in *Thalassia testidunum* meadows subjected to different phosphorus supplies: a lignin biomarker approach. *Limnol. Oceanogr*. 63 2630–2646. 10.1002/lno.10965

[B4] Baydoun E. A.-H. Brett C. T. (1985). Comparison of cell wall composition of the rhizomes of three seagrasses. *Aquat. Bot.* 23 191–196. 10.1016/0304-3770(85)90065-8

[B5] Bell D. J. Isherwood F. A. Hardwick N. E. (1954). D(+)-Apiose from the monocotyledon, *Posidonia australis*. *J. Chem. Soc.* 3702–3706.

[B6] Boerjan W. Ralph J. Baucher M. (2003). Lignin biosynthesis. *Annu. Rev. Plant Biol.* 54 519–546. 10.1146/annurev.arplant.54.031902.134938 14503002

[B7] Brennan M. Harris P. J. (2011). Distribution of fucosylated xyloglucans among the walls of different cell types in monocotyledons determined by immunofluorescence microscopy. *Mol. Plant* 4 144–156. 10.1093/mp/ssq067 20978085

[B8] Brudecki G. Farzanah R. Cybulska I. Schmidt J. E. Thomsen M. H. (2015). Evaluation of composition and biogas production potential from seagrasss (*Halodule uninervis*) native to Abu Dhabi. *Energy Procedia* 75 760–766. 10.1016/j.egypro.2015.07.508

[B9] Busse-Wicher M. Li A. Silveira R. L. Pereira C. S. Tryfona T. Gomes T. C. F. (2016). Evolution of xylan substitution patterns in gymnosperms and angiosperms: implications for xylan interaction with cellulose. *Plant Physiol.* 171 2418–2431. 10.1104/pp.16.00539 27325663 PMC4972281

[B10] Capanema E. A. Balakshin M. Y. Kadla J. F. (2005). Quantitative characterization of a hardwood milled wood lignin by nuclear magnetic resonance spectroscopy. *J. Agric. Food Chem.* 53 9639–9649. 10.1021/jf0515330 16332110

[B11] Chase M. W. Christenhusz M. J. M. Fay M. F. Byng J. W. Judd W. S. Soltis D. E. (2016). An update of the Angiosperm Phylogeny Group classification for the orders and families of flowering plants: APG IV. *Bot. J. Linn. Soc.* 181 1–20. 10.1111/boj.12385

[B12] Classen B. Baumann A. Utermoehlen J. (2019). Arabinogalactan-proteins in spore-producing land plants. *Carbohydr. Polym*. 210 215–224. 10.1016/j.carbpol.2019.01.077 30732757

[B13] Davey P. A. Pernice M. Sablok G. Larkum A. Lee H. T. Golicz A. (2016). The emergence of molecular profiling and omics techniques in seagrass biology; furthering our understanding of seagrasses. *Funct. Integr. Genomics* 16 465–480. 10.1007/s10142-016-0501-4 27443314

[B14] Davies P. Morvan C. Sire O. Baley C. (2007). Structure and properties of fibres from sea-grass (*Zostera marina*). *J. Mater. Sci.* 42 4850–4857. 10.1007/s10853-006-0546-1

[B15] Doblin M. S. Pettolino F. Bacic A. (2010). Plant cell walls: the skeleton of the plant world. *Funct. Plant Biol.* 37 357–381. 10.1071/FP09279

[B16] DuBois M. Gilles K. A. Hamilton J. K. Rebers P. A. Smith F. (1956). Colorimetric method for determination of sugars and related substances. *Anal. Chem.* 28 350–356. 10.1021/ac60111a017

[B17] Ducker S. C. Knox R. B. (1976). Submarine pollination in seagrasses. *Nature* 263 705–706. 10.1038/263705a0

[B18] Ducker S. C. Pettitt J. M. Knox R. B. (1978). Biology of Australian seagrasses: pollen development and submarine pollination in *Amphibolis antarctica* and *Thalassodendron ciliatum* (Cymodoceaceae). *Aust. J. Bot.* 26 265–285. 10.1071/BT9780265

[B19] Dupree R. Simmons T. J. Mortimer J. C. Patel D. Iuga D. Brown S. P. (2015). Probing the molecular architecture of *Arabidopsis thaliana* secondary cell walls using two-and three-dimensional ^13^C solid state nuclear magnetic resonance spectroscopy. *Biochemistry* 54 2335–2345. 10.1021/bi501552k 25739924

[B20] Ellis M. Egelund J. Schultz C. J. Bacic A. (2010). Arabinogalactan-Proteins: key regulators at the cell surface? *Plant Physiol.* 153 403–419. 10.1104/pp.110.156000 20388666 PMC2879789

[B21] Fry S. C. Nesselrode B. H. W. A. Miller J. G. Mewburn B. R. (2008). Mixed-linkage (1→3, 1→4)-β-D-glucan is a major hemicellulose of *Equisetum* (horsetail) cell walls. *New Phytol*. 179 104–115. 10.1111/j.1469-8137.2008.02435.x 18393951

[B22] Galiwango E. Abdel Rahman N. S. Al-Marzouqi A. H. Abu-Omar M. M. Khaleel A. A. (2019). Isolation and characterization of cellulose and α-cellulose from date palm biomass waste. *Heliyon* 5:e02937. 10.1016/j.heliyon.2019.e02937 32382665 PMC7201136

[B23] Gloaguen V. Brudieux V. Closs B. Barbat A. Krausz P. Sainte-Catherine O. (2010). Structural characterization and cytotoxic properties of an apiose-rich pectic polysaccharide obtained from the cell wall of the marine phanerogam *Zostera marina*. *J. Nat. Prod.* 73 1087–1092. 10.1021/np100092c 20465284

[B24] Hamdaoui O. Ibos L. Mazioud A. Safi M. Limam O. (2018). Thermophysical characterization of *Posidonia Oceanica* marine fibers intended to be used as an insulation material in Mediterranean buildings. *Constr. Build. Mater.* 180 68–76. 10.1016/j.conbuildmat.2018.05.195

[B25] Harris P. J. Webster J. Weinhandl J. A. Stone B. A. (1994). Composition of the walls of pollen grains of the seagrass *Amphibolis antarctica*. *Sex. Plant Reprod.* 7 101–106. 10.1007/BF00230578

[B26] Hatfield R. D. Rancour D. M. Marita J. M. (2016). Grass cell walls: a story of cross-linking. *Front. Plant Sci.* 7:2056. 10.3389/fpls.2016.02056 28149301 PMC5241289

[B27] He C. Zhang J. Liu X. Zeng S. Wu K. Yu Z. (2015). Identification of genes involved in biosynthesis of mannan polysaccharides in *Dendrobium officinale* by RNA-seq analysis. *Plant Mol. Biol*. 88 219–231. 10.1007/s11103-015-0316-z 25924595

[B28] Hemminga M. A. Duarte C. M. eds (2000). “Seagrasses in the human environment,” in *Seagrass Ecology* (Cambridge: Cambridge University Press), 248–291.

[B29] Herburger K. Holzinger A. (2015). Localization and quantification of callose in the streptophyte green algae *Zygnema* and *Klebsormidium*: correlation with desiccation tolerance. *Plant Cell Physiol*. 56 2259–2270. 10.1093/pcp/pcv139 26412780 PMC4650865

[B30] Hsieh Y. S. Y. Harris P. J. (2019). Xylans of red and green algae: what is known about their structures and how they are synthesised*?* *Polymers* 11:354. 10.3390/polym11020354 30960338 PMC6419167

[B31] Johnson K. L. Jones B. J. Bacic A. Schultz C. J. (2003). The fasciclin-like arabinogalactan proteins of *Arabidopsis*. A multigene family of putative cell adhesion molecules. *Plant Physiol.* 133 1911–1925. 10.1104/pp.103.031237 14645732 PMC300743

[B32] Kaal J. Serrano O. Del Río J. C. Rencoret J. (2018). Radically different lignin composition in *Posidonia* species may link to differences in organic carbon sequestration capacity. *Org. Geochem.* 124 247–256. 10.1016/j.orggeochem.2018.07.017

[B33] Kannan R. R. R. Arumugam R. Anantharaman P. (2013). Pharmaceutical potential of a fucoidan-like sulphated polysaccharide isolated from *Halodule pinifolia*. *Int. J. Biol. Macromol*. 62 30–34. 10.1016/j.ijbiomac.2013.08.005 23962716

[B34] Khiari R. Mhenni M. F. Belgacem M. N. Mauret E. (2010). Chemical composition and pulping of date palm rachis and *Posidonia oceanica*–a comparison with other wood and non-wood fibre sources. *Bioresour. Technol.* 101 775–780. 10.1016/j.biortech.2009.08.079 19766481

[B35] Khotimchenko M. Y. Lenskaya K. V. Petrakova M. Y. Khotimchenko Y. S. Kovalev V. V. (2006). The mercury binding activity of pectin isolated from the seagrass *Zostera marina*. *Russ. J. Mar. Biol*. 32:312 10.1134/S1063074006050099

[B36] Khotimchenko Y. Khozhaenko E. Kovalev V. Khotimchenko M. (2012). Cerium binding activity of pectins isolated from the seagrasses *Zostera marina* and *Phyllospadix iwatensis*. *Mar. Drugs* 10 834–848. 10.3390/md10040834 22690146 PMC3366678

[B37] Khozhaenko E. Kovalev V. Podkorytova E. Khotimchenko M. (2016). Removal of the metal ions from aqueous solutions by nanoscaled low molecular pectin isolated from seagrass *Phyllospadix iwatensis*. *Sci. Total Environ*. 565 913–921. 10.1016/j.scitotenv.2016.01.108 26848015

[B38] Klap V. A. Hemminga M. A. Boon J. J. (2000). Retention of lignin in seagrasses: angiosperm that returned to the sea. *Mar. Ecol. Prog. Ser*. 194 1–11. 10.3354/meps194001

[B39] Kolsi R. B. A. Fakhfakh J. Krichen F. Jribi I. Chiarore A. Patti F. P. (2016). Structural characterization and functional properties of antihypertensive *Cymodocea nodosa* sulfated polysaccharide. *Carbohydr. Polym*. 151 511–522. 10.1026/j.carbpol.2016.05.098 27474595

[B40] Lahaye P. A. Epstein E. (1969). Salt toleration by plants. Enhancement with calcium. *Science* 166 395–396. 10.1126/science.166.3903.395 17796555

[B41] Lamport D. T. A. Kieliszewski M. J. Showalter A. M. (2006). Salt stress upregulates periplasmic arabinogalactan proteins: using salt stress to analyse AGP function. *New Phytol.* 169 479–492. 10.1111/j.1469-8137.2005.01591.x 16411951

[B42] Lamport D. T. A. Tan L. Held M. Kieliszewski M. J. (2020). Phyllotaxis turns over a new leaf–A new hypothesis. *Int. J. Mol. Sci.* 21:1145. 10.3390/ijms21031145 32050457 PMC7037126

[B43] Lampugnani E. R. Khan G. A. Somssich M. Persson S. (2018). Building a plant cell wall at a glance. *J. Cell Sci*. 131:jcs207373. 10.1242/jcs.207373 29378834

[B44] Lee H. T. Golicz A. A. Bayer P. E. Jiao Y. Tang H. Paterson A. H. (2016). The genome of a southern hemisphere seagrass species (*Zostera muelleri*). *Plant Physiol.* 172 272–283. 10.1104/pp.16.00868 27373688 PMC5074622

[B45] Lee H. T. Golicz A. A. Bayer P. E. Severn-Ellis A. A. Chan C.-K. K. Batley J. (2018). Genomic comparison of two independent seagrass lineages reveals habitat-driven convergent evolution. *J. Exp. Bot.* 69 3689–3702. 10.1093/jxb/ery147 29912443 PMC6022596

[B46] Les D. H. Tippery N. P. (2013). “In time and with water.the systematics of alismatid monocotyledons,” in *Early Events in Monocot Evolution*, eds Wilkin P. Mayo S. J. (Cambridge: Cambridge University Press), 118–164.

[B47] Lewis N. G. Yamamoto E. (1990). Lignin: occurrence, biogenesis and biodegradation. *Annu. Rev. Plant Physiol. Plant Mol. Biol.* 41 455–496. 10.1146/annurev.pp.41.060190.002323 11543592

[B48] Li X. Jackson P. Rubtsov D. V. Faria-Blanc N. Mortimer J. C. Turner S. R. (2013). Development and application of a high throughput carbohydrate profiling technique for analyzing plant cell wall polysaccharides and carbohydrate active enzymes. *Biotechnol. Biofuels* 6:94. 10.1186/1754-6834-6-94 23819705 PMC3717103

[B49] Lv Y. Shan X. Zhao X. Cai C. Zhao X. Lang Y. (2015). Extraction, isolation, structural characterization and anti-tumor properties of an apigalacturonan-rich polysaccharide from the sea grass *Zostera caespitosa* Miki. *Mar. Drugs* 13 3710–3731. 10.3390/md13063710 26110894 PMC4483652

[B50] Ma Y. Zeng W. Bacic A. Johnson K. (2018). “AGPs through time and space,” in *Annual Plant Reviews online*, ed. Roberts J. A. (Hoboken, NJ: John Wiley and Sons).

[B51] Maeda M. Koshikawa M. Nisizawa K. Takano K. (1966). Cell wall constituents, especially pectic substance of a marine phanerogam *Zostera marina*. *Bot. Mag. Tokyo* 79 422–426. 10.15281/jplantres1887.79.422

[B52] Martone P. T. Estevez J. M. Lu F. Ruel K. Denny M. W. Somerville C. (2009). Discovery of lignin in seaweed reveals convergent evolution of cell-wall architecture. *Curr. Biol.* 19 169–175. 10.1016/j.cub.2008.12.031 19167225

[B53] Meikle P. J. Bonig I. Hoogenraad N. J. Clarke A. E. Stone B. A. (1991). The location of (1→3)-β-glucan-specific monoclonal antibody. *Planta* 185 1–8. 10.1007/BF00194507 24186272

[B54] Miroshnikov V. I. (1940). *Zostera* as an industrial raw material. *Zh. Prikl. Khim*. 13 1477–1489.

[B55] Mohnen D. (2008). Pectin structure and biosynthesis. *Curr. Opin. Plant Biol*. 11 266–277. 10.1016/j.pbi.2008.03.006 18486536

[B56] Moreira L. R. S. Filho E. X. F. (2008). An overview of mannan structure and mannan-degrading enzyme systems. *Appl. Microbiol. Biotechnol*. 79 165–178. 10.1007/s00253-008-1423-4 18385995

[B57] Moubasher M. H. Abdel-Hafez S. H. Mohanram A. A. (1982). Direct estimation of cellulose, hemicellulose, lignin. *J. Agric. Res.* 46 1467–1476.

[B58] Mukherjee A. Mandal T. Ganguly A. Chatterjee P. K. (2016). Lignin degradation in the production of bioethanol–A review. *ChemBioEng Rev.* 3 86–96. 10.1002/cben.201500016

[B59] National Center for Biotechnology Information (NCBI) (2020). *Glycans Page.* Available online at: www.ncbi.nlm.nih.gov/glycans/snfg.html (accessed September 22, 2020).

[B60] Ncibi M. C. Hamissa A. M. B. Gaspard S. (2014). “Plantae and marine biomass for biofuels,” in *Biomass for Sustainable Applications: Pollution Remediation and Energy*, eds Gaspard S. Ncibi M. C. (Cambridge: RSC Publishing), 290–334.

[B61] Ngo D.-H. Kim S.-K. (2013). Sulfated polysaccharides as bioactive agents from marine algae. *Int. J. Biol. Macromol.* 62 70–75. 10.1016/j.ijbiomac.2013.08.036 23994790

[B62] Olsen J. L. Rouzé P. Verhelst B. Lin Y.-C. Bayer T. Collen J. (2016). The genome of the seagrass *Zostera marina* reveals angiosperm adaptation to the sea. *Nature* 530 331–338. 10.1038/nature16548 26814964

[B63] Opsahl S. Benner R. (1993). Decomposition of senescent blades of the seagrass *Halodule wrightii* in a subtropical lagoon. *Mar. Ecol. Prog. Ser.* 94 191–205. 10.3354/meps094191

[B64] O’Rourke C. Gregson T. Murray L. Sadler I. H. Fry S. C. (2015). Sugar composition of the pectic polysaccharides of charophytes, the closest algal relatives of land-plants: presence of 3-O-methyl-D-galactose residues. *Ann. Bot.* 116 225–236. 10.1093/aob/mcv089 26113633 PMC4512192

[B65] Ovodov Y. S. Mikheyskaya L. V. Ovodova R. G. Krasikova I. N. (1971a). The pectic substances of *Zosteraceae*: part V. Smith degradation of zosterine. *Carbohydr. Res*. 18 319–322. 10.1016/S0008-6215(00)80356-0

[B66] Ovodov Y. S. Ovodova R. G. Bondarenko O. D. Krasikova I. N. (1971b). The pectic substances of *Zosteraceae*: part IV. Pectinase digestion of zosterine. *Carbohydr. Res*. 18 311–318. 10.1016/S0008-6215(00)80355-9

[B67] Ovodov Y. S. Ovodova R. G. Shibaeva V. I. Mikheyskaya L. V. (1975). Further structural studies of zosterine. *Carbohydr. Res.* 42 197–199. 10.1016/S0008-6215(00)84117-8

[B68] Ovodova R. G. Vaskovsky V. E. Ovodov Y. S. (1968). The pectic substances of *zosteraceae*. *Carbohydr. Res.* 6 328–332. 10.1016/S0008-6215(00)81454-8

[B69] Painter T. J. (1983). “4-Algal polysaccharides,” in *The Polysaccharides*, ed. Aspinall G. O. (Cambridge: Academic Press), 195–285.

[B70] Pavlakis L. (2018). Environmentally friendly panel from the dead seagrass leaves of *Posidonia oceanica*. International Patent PCT/GR2017/000043

[B71] Peña M. J. Kulkarni A. R. Backe J. Boyd M. O’Neill M. A. York W. S. (2016). Structural diversity of xylans in the cell walls of monocots. *Planta* 244 589–606. 10.1007/s00425-016-2527-1 27105886

[B72] Penning B. W. McCann M. C. Carpita N. C. (2019). Evolution of the cell wall families of Grasses. *Front. Plant Sci.* 10:1205. 10.3389/fpls.2019.01205 31681352 PMC6805987

[B73] Peralta A. G. Venkatachalam S. Stone S. C. Pattathil S. (2017). Xylan epitope profiling: an enhanced approach to study organ development-dependent changes in xylan structure, biosynthesis, and deposition in plant cell walls. *Biotechnol. Biofuels* 10:245. 10.1186/s13068-017-0935-5 29213310 PMC5707906

[B74] Petkowicz C. L. O. Reicher F. Chanzy H. Taravel F. R. Vuong R. (2001). Linear mannan in the endosperm of *Schizolobium amazonicum*. *Carbohydr. Polym*. 44 107–112. 10.1016/S0144-8617(00)00212-5

[B75] Pettit J. M. Jermy A. C. (1975). Pollen in hydrophilous angiosperms. *Micron* 5 377–405. 10.1016/0047-7206(74)90023-5

[B76] Pettitt J. M. (1980). Reproduction in seagrasses: nature of the pollen and receptive surface of the stigma in the Hydrocharitaceae. *Ann. Bot*. 45 257–271. 10.1093/oxfordjournals.aob.a085822

[B77] Pfeifer L. Shafee T. Johnson K. Bacic T. Classen B. (2020). Arabinogalactan-proteins of *Zostera marina* L. contain unique glycan structures and provide insight into adaption processes to saline environments. *Sci. Rep.* 10:8232. 10.1038/s41598-020-65135-5 32427862 PMC7237498

[B78] Pomin V. H. (2012). “Chapter 12–structure–function relationship of anticoagulant and antithrombotic well-defined sulfated polysaccharides from marine invertebrates,” in *Marine Medical Foods: Implications and Applications–Animals and Microbes*, ed. Se-Kwon K. (Amsterdam: Academic Press), 195–209. 10.1016/B978-0-12-416003-3.00012-3 22361188

[B79] Popov S. Popova G. Golovchenko V. Ovodova R. (2007). Preventative anti-inflammatory effect of pectic galacturonans after oral administration. *Planta Med.* 73:P–015. 10.1055/s-2007-986797

[B80] Popper Z. A. Fry S. C. (2004). Primary cell wall composition of pteridophytes and spermatophytes. *New Phytol*. 164 165–174. 10.1111/j.1469-8137.2004.01146.x 33873476

[B81] Popper Z. A. Fry S. C. (2008). Xyloglucan – pectin linkages are formed intra-protoplasmatically, contribute to wall-assembly, and remain stable in the cell wall. *Planta* 227 781–794. 10.1007/s00425-007-0656-2 17987313

[B82] Raimundo S. C. Avci U. Hopper C. Pattathil S. Hahn M. G. Popper Z. A. (2016). Immunolocalization of cell wall carbohydrate epitopes in seaweeds: presence of land plant epitopes in *Fucus vesiculosus* L. (Phaeophyceae). *Planta* 243 337–354. 10.1007/s00425-015-2412-3 26411728

[B83] Sablok G. Hayward R. J. Davey P. A. Santos R. P. Schliep M. Larkum A. (2018). SeagrassDB: an open-source transcriptomics landscape for phylogenetically profiled seagrasses and aquatic plants. *Sci. Rep*. 6:2749. 10.1038/s41598-017-18782-0 29426939 PMC5807536

[B84] Saijonkari-Pahkala K. (2008). Non-wood plants as raw material for pulp and paper. *Agr. Food Sci.* 10:101 10.23986/afsci.5707

[B85] Salmean A. A. Duffieux D. Harholt J. Qin F. Michel G. Czjek M. (2017). Insoluble (1→3), (1→4)-β-D-glucan is a component of cell walls in brown algae (*Phaeophyceae*) and is masked by alginates in tissues. *Sci. Rep*. 7:2880. 10.1038/s41598-017-03081-5 28588313 PMC5460208

[B86] Serrano O. Rozaimi M. Lavery P. S. Smernik R. J. (2020). Organic chemistry insights for the exceptional soil carbon storage of the seagrass *Posidonia australis*. *Estuar. Coast. Shelf Sci.* 237:106662 10.1016/j.ecss.2020.106662

[B87] Silva J. M. C. Dantas-Santos N. Gomes D. L. Costa L. S. Cordeiro S. L. Costa M. S. S. P. (2012). Biological activities of the sulfated polysaccharide from the vascular plant *Halodule wrightii*. *Rev. Bras. Farmacogn.* 22 94–101. 10.1590/S0102-695X2011005000199

[B88] Smith M. M. McCully M. E. (1978). A critical evalutation of the specifity of aniline blue induced fluorescence. *Protoplasma* 95 229–254. 10.1007/BF01294453

[B89] Stone B. Clarke A. E. (1992). *Chemistry and Biology of (1→3)-[Beta]-Glucans.* Bundoora, VIC: La Trobe University Press.

[B90] Strasser R. (2014). Biological significance of complex *N*-glycans in plants and their impact on plant physiology. *Front. Plant Sci.* 5:363. 10.3389/fpls.2014.00363 25101107 PMC4105690

[B91] Syed N. N. F. Zakaria M. H. Bujang J. S. (2016). Fiber characteristics and papermaking of seagrass using Hand-beaten and blended pulp. *Bioresources* 11 5358–5380. 10.15376/biores.11.2.5358-5380

[B92] The Plant List (2020). *Ruppia.* Available online at: http://www.theplantlist.org/1.1/browse/A/Ruppiaceae/Ruppia/ (accessed September 22, 2020).

[B93] Torbatinejad N. M. Annison G. Rutherfurd-Markwick K. Sabine J. R. (2007). Structural constituents of the seagrass *Posidonia australis*. *J. Agric. Food Chem.* 55 4021–4026. 10.1021/jf063061a 17439231

[B94] Torbatinejad N. M. Sabine J. R. (2001). Laboratory evaluation of some marine plants on South Australian Beaches. *J. Agric. Sci. Technol*. 3 91–100.

[B95] Tryfona T. Sorieul M. Feijao C. Stott K. Rubtsov D. V. Anders N. (2019). Development of an oligosaccharide library to characterize the structural variation in glucuronoarabinoxylan in the cell walls of vegetative tissues in grasses. *Biotechnol. Biofuels* 12:109. 10.1186/s13068-019-1451-6 31080516 PMC6501314

[B96] van Erven G. de Visser R. Merkx D. W. H. Strolenberg W. de Gijsel P. Gruppen H. (2017). Quantification of lignin and ist structural features in plant biomass using ^13^C lignin as internal standard for pyrolysis-GC-SIM-MS. *Anal. Chem.* 89 10907–10916. 10.1021/acs.analchem.7b02632 28926698 PMC5647568

[B97] Verma D. P. S. Hong Z. (2001). Plant callose synthase complexes. *Plant Mol. Biol.* 47 693–701. 10.1023/A:1013679111111 11785931

[B98] Waldron K. W. Baydoun E. A.-H. Brett C. T. (1989). Comparison of cell wall composition of tissues from the seagrasses *Halophila* and *Halodule*. *Aquat. Bot.* 35 209–218.

[B99] Webster J. Stone B. A. (1994). Isolation, histochemistry and monosaccharide composition of the walls of root hairs from *Heterozostera tasmanica* (Martens ex Aschers.) den Hartog. *Aquat. Bot.* 39–52. 10.1016/0304-3770(94)90047-7

[B100] Wefers D. Bunzel M. (2016). NMR spectroscopic profiling of arabinan and galactan Structural Elements. *J. Agric. Food Chem*. 64 9559–9568. 10.1021/acs.jafc.6b04232 27936685

[B101] Weng J.-K. Chapple C. (2010). The origin and evolution of lignin biosynthesis. *New Phytol*. 187 273–285. 10.1111/j.1469-8137.2010.03327.x 20642725

[B102] Willats W. G. T. McCartney L. Mackie W. Knox J. P. (2001). Pectin: cell biology and prospects for functional analysis. *Plant Mol. Biol.* 47 9–27. 10.1023/A:1010662911148 11554482

[B103] Williams S. L. (2016). From sea to sea. *Nature* 530 290–291. 10.1038/nature16869 26814973

[B104] Wissler L. Dattolo E. Moore A. D. Reusch T. B. H. Olsen J. L. Migliaccio M. (2009). Dr. Zompo: an online data repository for *Zostera marina* and *Posidonia oceanica* ESTs. *Database* 2009:bap009. 10.1093/database/bap009 20157482 PMC2790305

[B105] Woolard G. R. Jones J. K. N. (1978). Polysaccharides of the sea grass *Phyllospadix torreyi*. *Carbohydr. Res.* 63 327–332. 10.1016/S0008-6215(00)80964-7

[B106] Wu S.-W. Kumar R. Iswanto A. B. B. Jae-Yean Kim J.-Y. (2018). Callose balancing at plasmodesmata. *J. Exp. Bot.* 69 5325–5339. 10.1093/jxb/ery317 30165704

[B107] Wyllie-Echeverria S. Cox P. A. (1999). The seagrass (*Zostera marina* [Zosteraceae]) industry of Nova Scotia (1907-1960). *Econ. Bot.* 53:419 10.1007/BF02866721

[B108] York W. S. O’Neill M. A. (2008). Biochemical control of xylan biosynthesis – which end is up? *Curr. Opin. Plant Biol.* 11 258–265. 10.1016/j.pbi.2008.02.007 18374624

[B109] Yoshiie T. Maeda M. Kimura M. Hama Y. Uchida M. Kimura Y. (2012). Structural features of *N*-Glycans of seaweed glycoproteins: predominant occurrence of High-Mannose Type *N-*glycans in marine plants. *Biosci. Biotech. Bioch.* 76 1996–1998. 10.1271/bbb.120463 23047116

[B110] Zidorn C. (2016). Secondary metabolites of seagrasses (Alismatales and Potamogetonales; Alismatidae): chemical diversity, bioactivity, and ecological function. *Phytochemistry* 214 5–28. 10.1016/j.phytochem.2016.02.004 26880288

